# Ubiquitin conjugating enzyme E2 L3 promoted tumor growth of NSCLC through accelerating p27kip1 ubiquitination and degradation

**DOI:** 10.18632/oncotarget.20449

**Published:** 2017-08-24

**Authors:** Xingjie Ma, Junjie Zhao, Fan Yang, Haitao Liu, Weibo Qi

**Affiliations:** ^1^ Department of Cardiothoracic Surgery, The First Affiliated Hospital of Jiaxing University, Jiaxing, Zhejiang, China

**Keywords:** NSCLC, UBE2L3, p27kip1, ubiquitination, degradation

## Abstract

The molecular pathogenesis of human lung cancer has not been completely clarified. Here, we reported that UBE2L3, a member of the ubiquitin-conjugating enzymes (E2s), were overexpressed in non-small-cell lung cancer (NSCLC) tissues compared with the non-tumor tissues. High expression of UBE2L3 was correlated with advanced tumor stage and adverse outcomes. Knockdown of UBE2L3 inhibited NSCLC cell growth while ectopic expression of UBE2L3 promoted NSCLC cell growth in a cell cycle dependent manner. The results of subcutaneous tumor xenograft studies revealed that knockdown of UBE2L3 attenuated the *in vivo* tumor growth. Mechanistically, we observed that UBE2L3 could interact with F-box protein Skp2, a member of the SCF (Skp2) ubiquitin ligase complex, and thus promoted the ubiquitination and proteasomal degradation of p27kip1. Furthermore, NSCLC cases with high level of UBE2L3 and low level of p27kip1 had worst prognosis, suggesting that combination of UBE2L3 and p27kip1 is a more powerful prognostic marker for NSCLC patients. Taken together, the current study presented a novel marker for predicting prognosis and a potential therapeutic target for NSCLC patients.

## INTRODUCTION

Lung cancer remains the leading cause of cancer death worldwide with a 5-year survival rate less than 15 % [1, 2]. Non-small-cell lung cancer (NSCLC) is the most common type of lung cancer and its molecular pathogenesis has not been completely clarified. Emerging evidences have indicated that post-translational modifications, including ubiquitination, phosphorylation, and methylation of tumor progression related proteins were involved in the carcinogenesis of NSCLC, implicating a potential to develop novel prognostic biomarkers and therapeutic strategies for this deadly disease, which still need further investigations [3–5].

The ubiquitin-protease system (UPS) is a complex multi-stage enzyme cascade through which proteins are labelled with ubiquitin and recognized by proteasomes for degradation [6, 7]. Generally, degradation of target proteins is a collaborative work by three kinds of enzymes: ubiquitin-activating enzyme (E1), which could attach to the lysine residues of ubiquitin for activation; ubiquitin conjugating enzyme (E2), which could transfer ubiquitin to the lysine residues of target proteins; ubiquitin ligase (E3), which is mainly responsible for the identification and ubiquitination of target proteins [8, 9]. To the end, ubiquitin-tagged proteins are degraded into smaller polypeptides, amino acids and ubiquitin that can be reused. Ubiquitination is involved in lots of biological process including cell cycle, proliferation, apoptosis, differentiation, DNA repair, inflammation and immunity [9–11]. As to human tumors, ubiquitin mediated proteolysis controls the stability of tumor suppressor proteins and oncogene products. For instance, overexpression of several E3 ubiquitin ligases could breach the stability of tumor suppressor proteins including p53, p21cip1 and p27kip1 [12, 13]. However, little is known about the potential contribution of E2 ubiquitin conjugating enzyme to carcinogenesis. The clinical expression or biological significance of ubiquitin conjugating enzyme E2 L3 (UBE2L3) was rarely reported in human malignancies. A recent literature revealed that UBE2L3 could regulate the stability of tumor suppressor p53-binding protein 1 (53BP1), which was an important anticancer target of human tumors [14]. UBE2L3 was also reported to be involved in the ubiquitination of c-Fos and the NF-kB precursor p105, suggesting the potential relationship between UBE2L3 and human tumors [15, 16]. In the current work, we investigated the clinical significance and biological function of UBE2L3 in NSCLC.

## RESULTS

### UBE2L3 is overexpressed in NSCLC tissues and correlates with poor prognosis of NSCLC patients

**Figure 1:**
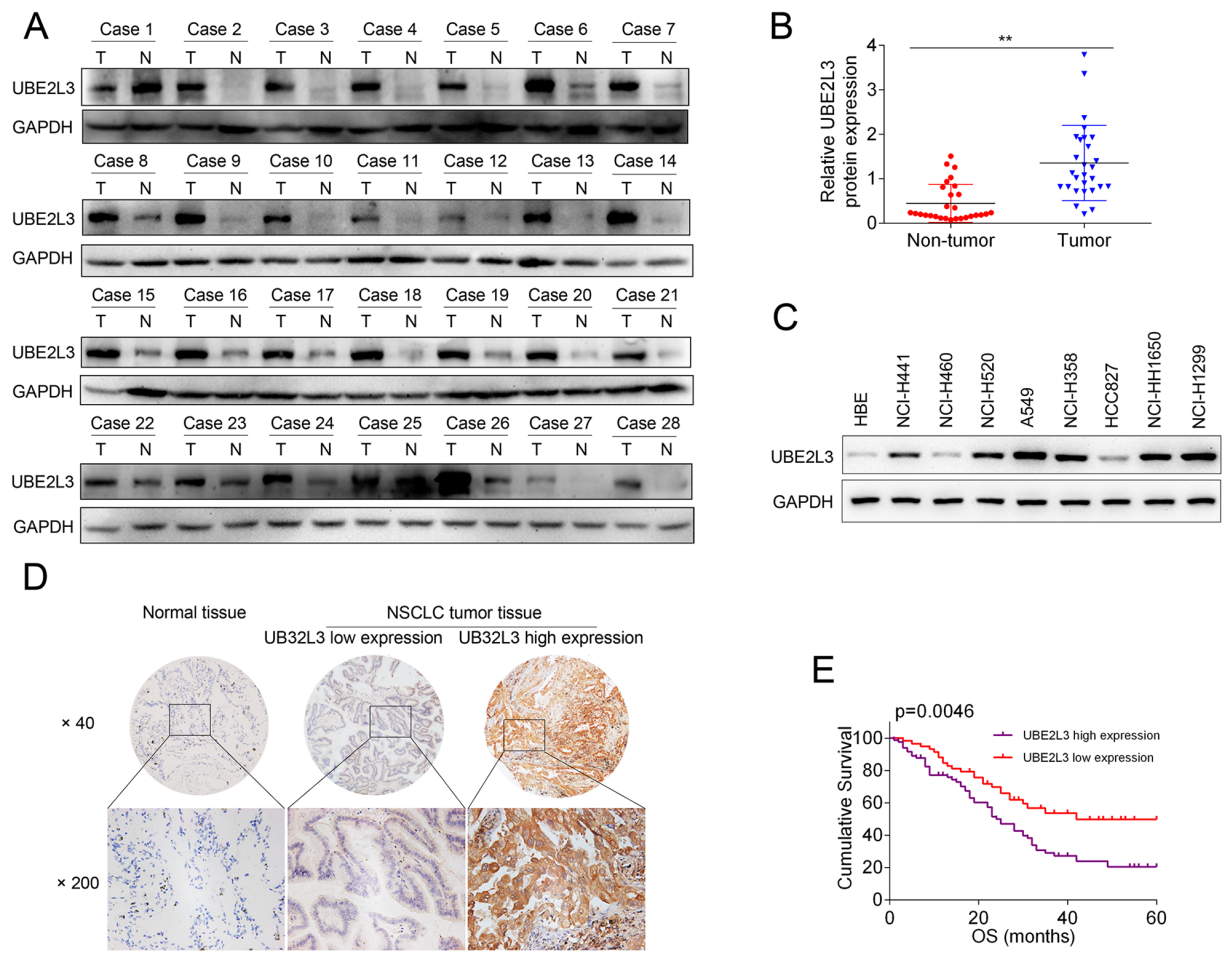
Expression of UBE2L3 and its clinical significance in NSCLC patients **(A)** Representativeimmunoblotting (IB) images of UBE2L3 expression in 28 paired NSCLC samples. **(B)** Quantification of relative grey value of UBE2L3 compared with GAPDH. **(C)** The protein level of UBE2L3 was detected by IB in eight NSCLC cell lines and the normal immortalized bronchial epithelial cell line HBE. **(D)** Representative images of the immunohistochemical staining for UBE2L3 in tumor tissues and non-tumor tissues. **(E)** NSCLC patients with high level of UBE2L3 showed worse overall survival compared with the cases exhibited low level of UBE2L3. (Data were presented with mean ± SD of three independent experiments, ^**^p<0.01).

To assess UBE2L3 expression in NSCLC, we first examined UBE2L3 level in 28 cases of NSCLC and matched non-tumor tissues by qRT-PCR and IB. Results revealed that UBE2L3 expression in NSCLC tissues was higher than that of non-tumor tissues, with a significant difference at the mRNA (Supplementary Figure 1A) and protein levels (Figure 1A and Figure 1B, *p*<0.01). Subsequently, we detected UBE2L3 expression in NSCLC by using immunohistochemistry technique combined with tissue microarray. In the 142 NSCLCs, high expression of UBE2L3 was observed in 83 (58.5 %) tumors (Figure 1D). The relationship between UBE2L3 expression and the clinicpathological characteristics in 142 NSCLC patients was listed in Table 1. Results demonstrated that high expression of UBE2L3 was correlated with advanced tumor stage, while no difference was observed between UBE2L3 level and other clinicpathological characteristics including gender, age, smoking, histopathology type as well as tumor differentiation. More importantly, the elevated UBE2L3 expression was closely associated with poorer survival of NSCLC as determined by the Kaplan-Meier and log-rank tests for survival analysis (Figure 1E).

**Table 1:** Relationship between UBE2L3 expression and clinicopathological characteristics of NSCLC cases (n = 142)

Parameters	n	UBE2L3 expression		
		Low	High	*p*
Total	142	59	83	
Age (years)				
<60	63	22	41	0.152
≥60	79	37	42	
Gender				
Male	108	43	65	0.455
Female	34	16	18	
Smoking				
No smoking	82	36	46	0.506
Smoking	60	23	37	
Histopathology type				
Adenocarcinoma	39	14	25	0.400
Squamous cell carcinoma	103	45	58	
TNM stage				
Stage I	37	26	11	<0.001
Stage II	58	21	37	
Stage III-IV	47	12	35	
Differentiation				
Low grade	17	9	8	0.099
Middle grade	90	38	52	
High grade	35	12	23	

### High levels of UBE2L3 are positively correlated with NSCLC cell growth *in vitro*

**Figure 2:**
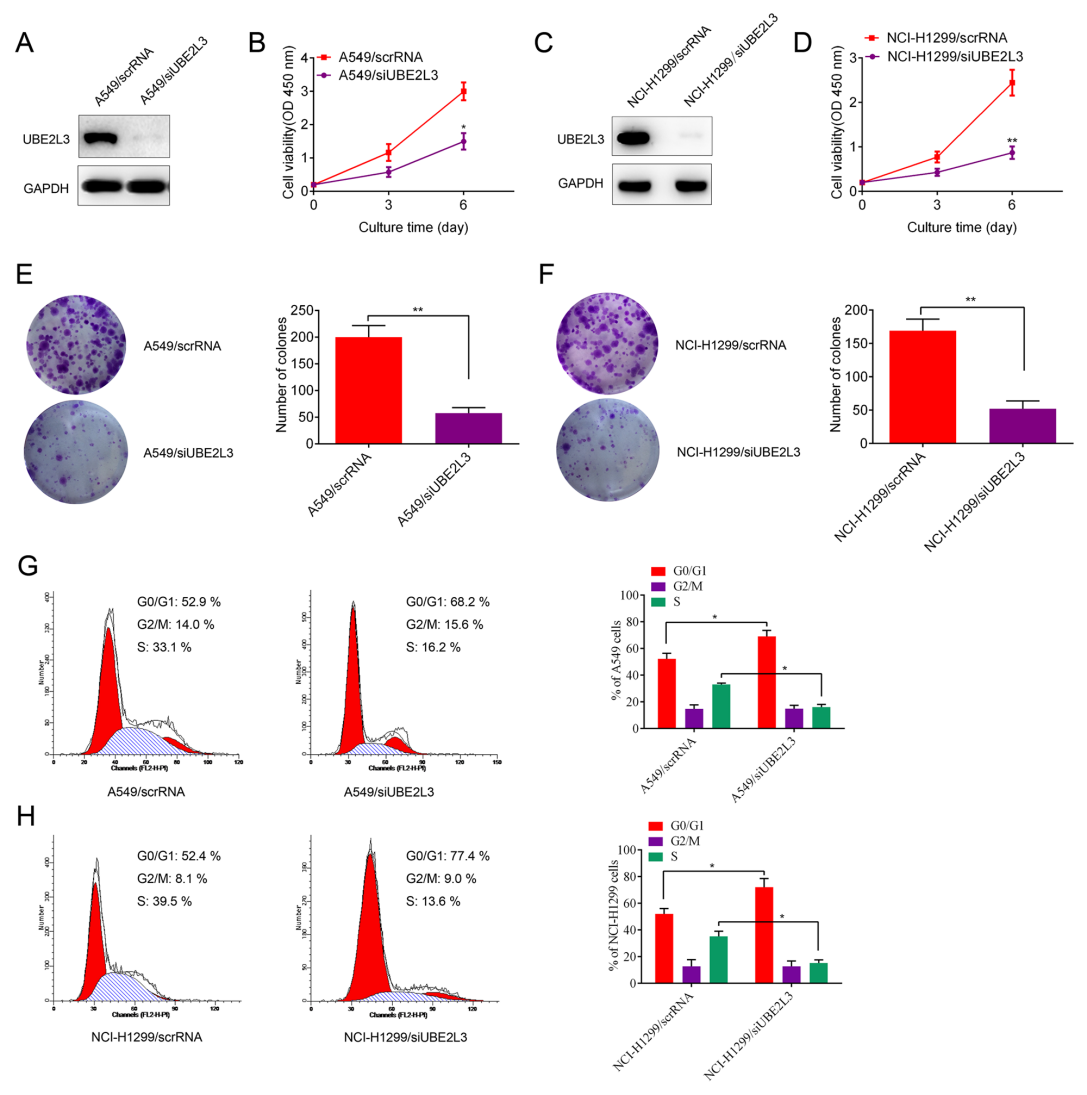
Knockdown of UBE2L3 inhibited cell cycle dependent proliferation of NSCLC cells **(A, C)** Knockdown efficiency of UBE2L3 in A549 and NCI-H1299 cells were confirmed by IB. **(B, D)** Knockdown of UBE2L3 inhibited cell proliferation of A549 and NCI-H1299 cells by CCK-8 assay. **(E, F)** Knockdown of UBE2L3 inhibited colony formation ability of A549 and NCI-H1299 cells. **(G, H)** Cell cycle of NSCLC cells was detected by flow cytometry and the results indicated that knockdown of UBE2L3 increased proportion of cells in G1 phase. (Data were presented with mean ± SD of three independent experiments, ^*^p<0.05, ^**^p<0.01).

We then investigated the functional role of UBE2L3 in promoting lung cancer progression. Firstly, we detected the expression of UBE2L3 in eight NSCLC cell lines and the normal immortalized bronchial epithelial cell line HBE by qRT-PCR and IB assay. The results revealed that UBE2L3 expression is highly diverse in NSCLC cell lines. Among these cell lines, A549 and NCI-H1299 cells showed high level of UBE2L3, whereas NCI-H460 and HCC827 cells expressed low level of UBE2L3 (**protein level:** Figure 1C; **mRNA level:** Supplementary Figure 1B). Then we asked whether UBE2L3 was involved in the regulation of NSCLC cell growth *in vitro*. Loss of function assay with siRNA was performed and transfection efficiency was evaluated by IB. As shown in Figure 2A to Figure 2F, transfecting A549 and NCI-H1299 cells with siUBE2L3 (A549/siUBE2L3 and NCI-H1299/siUBE2L3) significantly suppressed cell proliferation and colony formation ability compared with the control groups (A549/scrRNA and NCI-H1299/scrRNA). We also transfected UBE2L3 expressing plasmids into NCI-H460 and HCC827 cells with low UBE2L3 expression and found that overexpression of UBE2L3 promoted the proliferation and colony formation ability (Supplementary Figure 2A to Supplementary Figure 2E).

The effect of UBE2L3 on cell proliferation was further assessed by cell cycle and apoptosis analysis. As the result, UBE2L3 inhibition resulted in increased proportion of cells in G1 phase (*p*<0.05, Figure 2G and Figure 2H) and a slightly increased apoptosis rate (no significance, data not shown) of NSCLC cells while overexpression of UBE2L3 resulted in a decreased proportion of cells in G1 phase (*p*<0.05, Supplementary Figure 2F and Supplementary Figure 2G) and a slightly decreased apoptosis rate (no significance, data not shown). These data suggested UBE2L3 promoted *in vitro* NSCLC cell growth via affecting cell cycle distribution.

### UBE2L3 induces p27kip1 ubiquitination and proteasomal degradation in cooperation with E3 ubiquitin ligase Skp2

**Figure 3:**
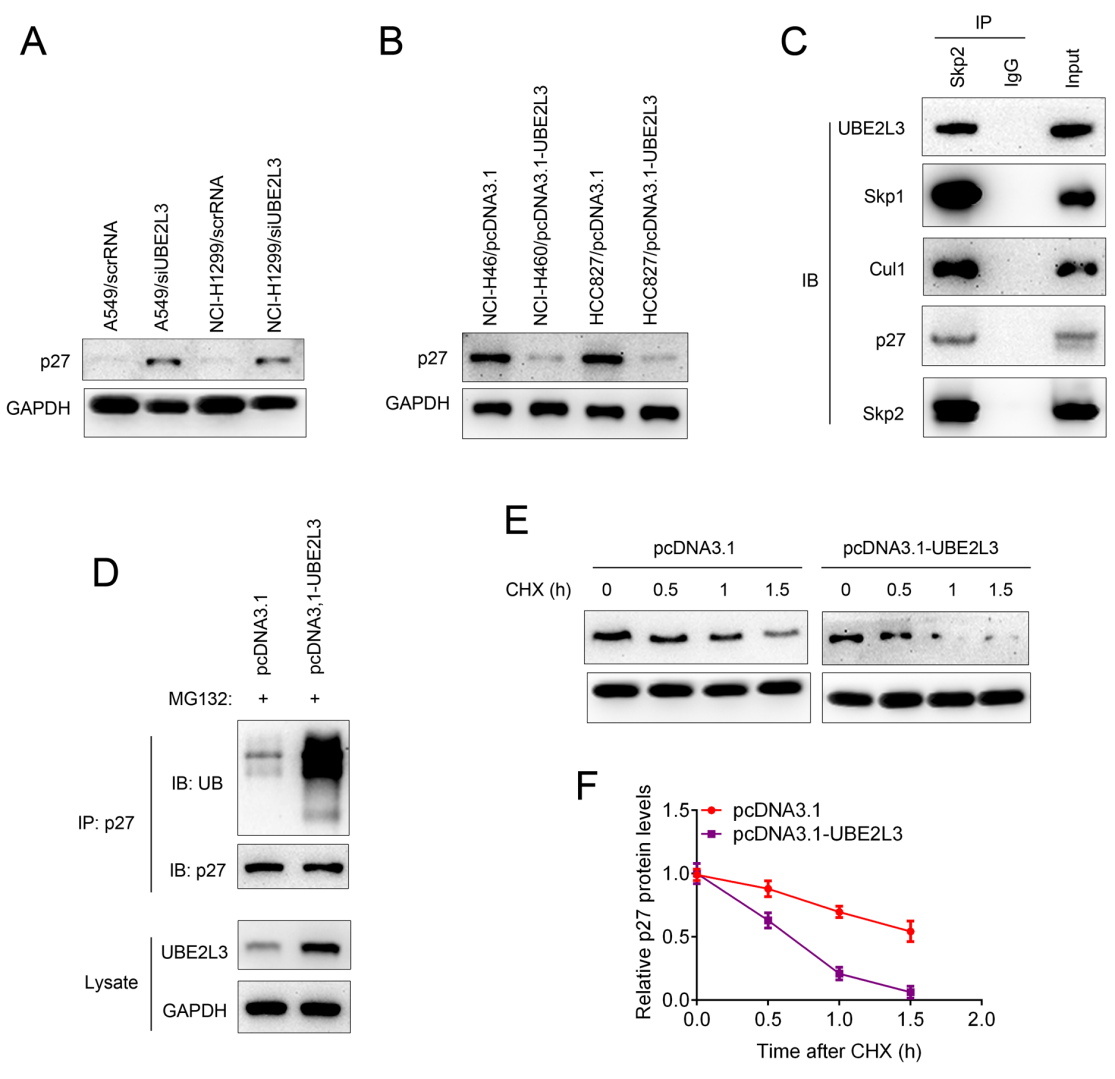
UBE2L3 interacted with SCF (Skp2) complex and promoted p27kip1 ubiquitination and proteasomal degradation **(A)** Knockdown of UBE2L3 in A549 and NCI-H1299 cells promoted the protein level of p27kip1. **(B)** Overexpression of UBE2L3 in NCI-H460 and HCC827 cells decreased the protein level of p27kip1. **(C)** The association between UBE2L3 and SCF (Skp2) complex and p27kip1 was confirmed by immunoprecipitation and immunoblotting assay. **(D)** Overexpression of UBE2L3 promoted the ubiquitination and proteasomal degradation of p27kip1. **(E, F)** The half-life of p27kip1 was evaluated by CHX assay after UBE2L3 overexpression.

Cyclin-dependent kinase inhibitor p27Kip1 could inhibit cell cycle transition from G1 phase to S phase and is degradated at late G1 phase. Accelerated degradation of p27kip1 was involved in the carcinogenesis of multiple human cancers. Since UBE2L3 functioned as an ubiquitin-specific protease and UBE2L3 could accelerate the cell cycle transition from G1 phase to S phase, we hypothesized UBE2L3 might regulate p27kip1 level in a proteasome-dependent manner. To this end, we first evaluated whether UBE2L3 could regulate p27kip1 expression. Co-localization of UBE2L3 and p27kip1 was presented in Supplementary Figure 3A while neither UBE2L3 overexpression nor down-regulation affected p27kip1 mRNA levels (Supplementary Figure 3B and Supplementary Figure 3C). Interestingly, our results showed that overexpression of UBE2L3 decreased the protein level of p27kip1, while knockdown of UBE2L3 promoted the protein level of p27kip1 (Figure 3A and Figure 3B). These results suggested that UBE2L3 might be involved in the post-translational regulation of p27kip1.

The F-box protein Skp2, a critical member of the Skp1-Cul1-F-box (SCF) E3 ubiquitin complex, was well reported to be involved in the ubiquitination and proteasomal degradation of p27kip1. In our current work, we observed that the SCF (Skp2) complex could interact with E2 ubiquitin-conjugating enzyme UBE2L3 as well as the target protein p27kip1 (Figure 3C). We then conducted loss-of-function assay of Skp2 to confirm the Skp2-p27kip1 axis. As indicated in Supplementary Figure 4A, knockdown of Skp2 promoted the level of p27kip1 in both cell lines. The Co-IP assay showed that knockdown of Skp2 inhibited the ubiquitination and proteasomal degradation of p27kip1 (Supplementary Figure 4B). Additionally, Figure 3D showed that overexpression of UBE2L3 promoted the poly-ubiquitination of p27kip1. These results suggested that UBE2L3 regulates p27kip1 level in a proteasome-dependent manner and drawing a relatively complete degradation pathway for p27kip1. To further evaluate whether UBE2L3 affected p27kip1 stability, we treated cells with CHX and determined the half-life of p27kip1. The results indicated that overexpression of UBE2L3 decreased the stability of p27kip1 (Figure 3E and Figure 3F). Taken together, UBE2L3 promoted the ubiquitination and proteasomal degradation of p27kip1 in cooperation with the SCF (Skp2) complex.

### UBE2L3 regulated NSCLC cell growth through p27kip1

**Figure 4:**
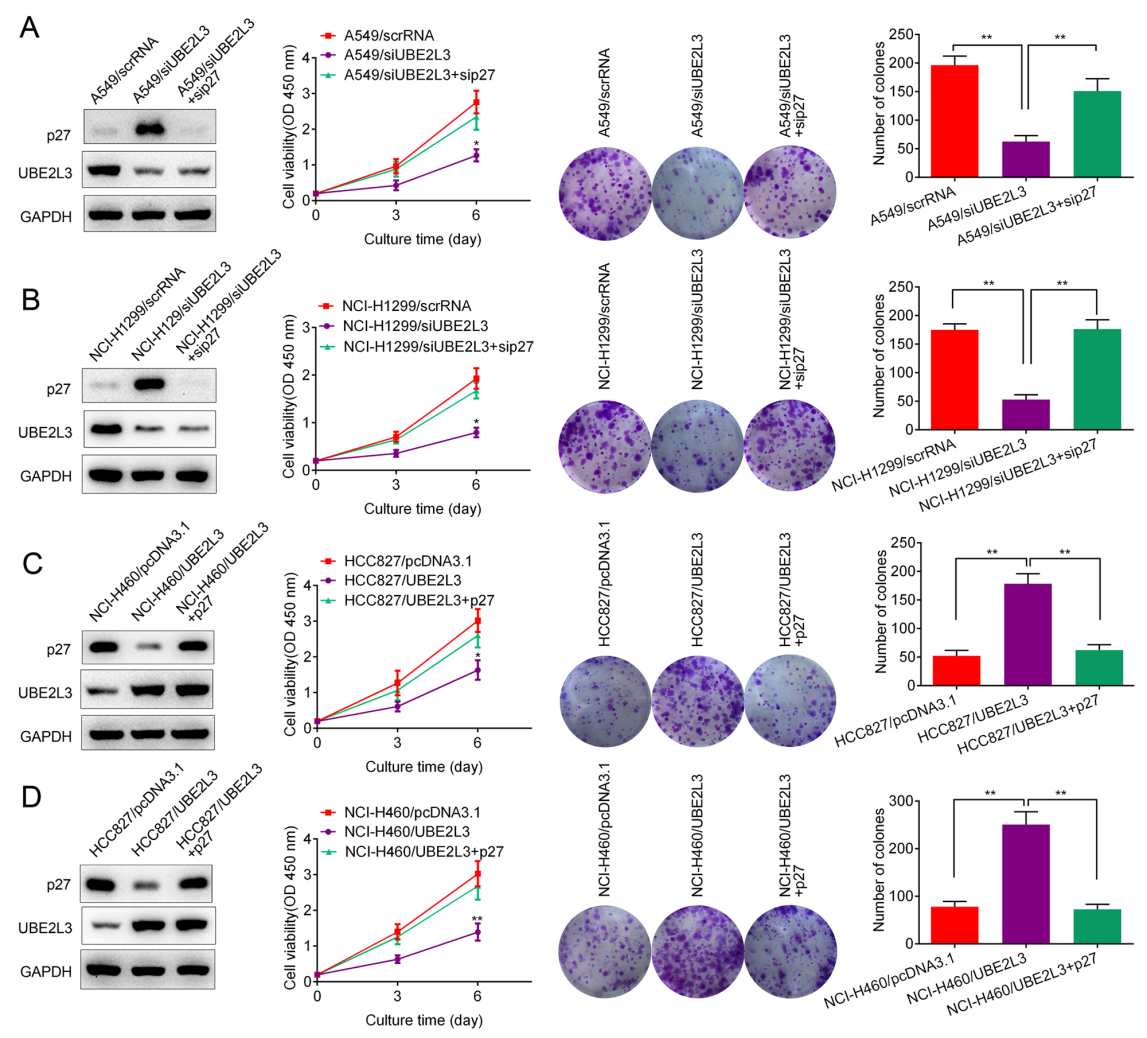
p27kip1 mediated UBE2L3's function in NSCLC cells **(A, B)** Knockdown of UBE2L3 in A549 and NCI-H1299 cells promoted the level of p27kip1 and inhibited NSCLC cell growth, while co-transfection of siUBE2L3 and sip27kip1 downregulated the level of p27kip1 and promoted NSCLC cell growth. **(C, D)** Overexpression of UBE2L3 decreased the level of p27kip1 and promoted NSCLC cell growth, while co-transfection of UBE2L3 and p27kip1 expressing plasmids promoted the level of p27kip1 and inhibited NSCLC cell growth. (Data were presented with mean ± SD of three independent experiments, ^**^p<0.01).

Given that p27kip1 was a downstream target of UBE2L3, we next wanted to know whether p27kip1 was responsible for UBE2L3 induced cell aggressiveness. As shown in Figure 4A and Figure 4B, cell proliferation and colony formation were rescued after A549 or NCI-H1299 cells were co-transfected with siUBE2L3 and sip27kip1. In addition, cell proliferation and colony formation were also rescued after NCI-H460 or HCC827 cells were co-transfected with UBE2L3 and p27kip1 expressing plasmids (Figure 4C and Figure 4D). These data indicated that UBE2L3 promoted NSCLC cell growth and through p27kip1.

### The prognostic value of combination of UBE2L3 and p27kip1

**Figure 5:**
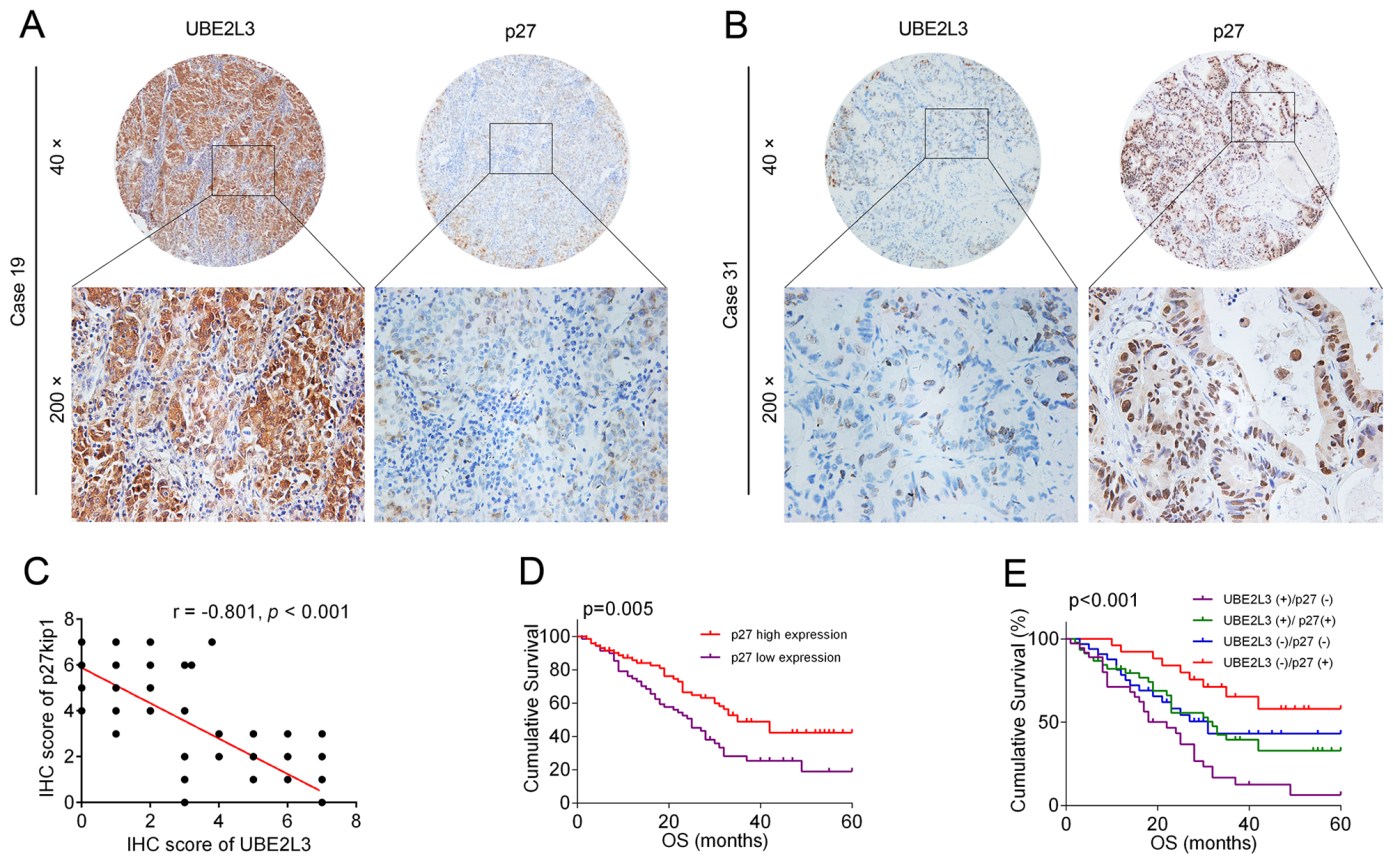
UBEL23 and p27kip1 could be used as prognostic biomarkers together **(A, B)** Representative immunohistochemicalimages of UBEL23 and p27kip1. **(C)** Correlation analysis of UBEL23 and p27kip1 expression were presented. **(D)** High level of p27kip1 predicted better overall survival (OS) of NSCLC patients. **(E)** NSCLC cases expressing high level of UBE2L3 and low level of p27kip1 had the poorest prognosis in terms of OS.

Given that UBE2L3 primarily functions in cancer initiation via accelerating p27kip1 degradation, we then asked whether the combination of UBE2L3 and p27kip1 could better predict survival than either protein. As the result, patients with low level of p27kip1 exhibited better prognosis than the others (Figure 5A and Figure 5B), suggesting p27kip1 might be served as a positive prognostic marker of NSCLC patients. Our results also showed that the expression of UBE2L3 was conversely related with p27kip1 in NSCLC samples (Pearson's correlation, r =-0.801, p < 0.01, Figure 5C). Moreover, NSCLC patients whose tumors expressing high levels of UBE2L3 and low levels of p27kip1 exhibited worst prognoses (Figure 5D and Figure 5E). These data provide evidences that UBE2L3 and p27kip1 can be used as prognostic markers together.

### UBE2L3 could regulate tumor growth *in vivo*

**Figure 6:**
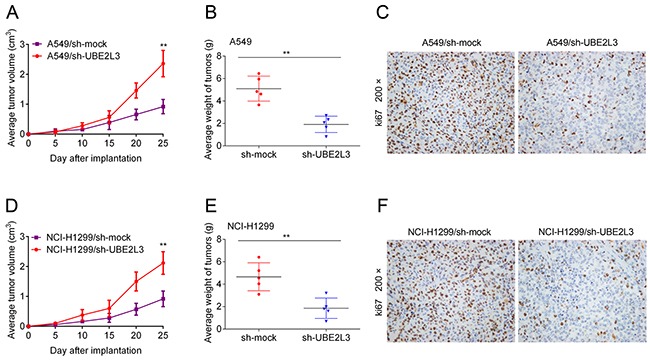
UBE2L3 contributes to NSCLC progression *in vivo* **(A, D)** A549 and NCI-H1299 cells stably expressing shUBE2L3 or control vector were subcutaneously transplanted into nude mice and the growth curves were shown. **(B, E)** The weights of tumors were presented as mean ± SD. **(C, F)** IHC staining demonstrated that knockdown of UBE2L3 inhibited NSCLC cell growth *in vivo*, as indicated by the expression of ki67. (Data were presented with mean ± SD of three independent experiments, ^**^p<0.01).

To determine whether UBE2L3 expression is necessary for the growth of xenograft tumors in mice, we used stable cell lines expressing UBE2L3 shRNA (shUBE2L3) or NC shRNA (shNC) to establish xenograft tumors in nude mice. As the result, knockdown of UBE2L3 significantly suppressed the growth of A549 (Figure 6A and Figure 6B) and NCI-H1299 (Figure 6D and Figure 6E) xenograft tumors. The results of IHC showed that Ki-67 positive cells of shUBE2L3 group were much less than shNC group (Figure 6C and Figure 6F). Additionally, subcutaneous xenografts derived from A549/shUBE2L3 or NCI-H1299/shUBE2L3 cells showed higher levels of p27kip1 compared with control groups (Supplementary Figure 4C). Altogether, knockdown of UBE2L3 inhibited *in vivo* tumor growth of NSCLC cells.

## DISCUSSION

Recently, E2 ubiquitin conjugating enzyme UBE2C was reported to be overexpressed in multiple human tumors and might be served as a potential cancer biomarker [17, 18]. In NSCLC, overexpression of UBE2C in tumor tissues was correlated with advanced tumor stage and apoptosis dependent cell proliferation [19]. Another study in breast cancer indicated that UBE2C down-regulation promoted the chemosensitivity of epirubicin and docetaxel resistant breast cancer cells [20]. However, there still lack a comprehensive analysis between UBE2L3 and lung cancer. In the current work, we found the mRNA and protein levels of UBE2L3 were up-regulated in NSCLC tissues in comparison with non-tumor tissues. In addition, UBE2L3 was also found to be overexpressed in a series of NSCLC cell lines compared with the normal immortalized bronchial epithelial cell line HBE. These results drove us to further evaluate the clinical significance of UBE2L3 in NSCLC. By scoring the result of immunohistochemistry, we got a significant relationship between UBE2L3 level and the TNM stage of NSCLC patients. Furthermore, overexpression of UBE2L3 also predicted adverse outcomes of NSCLC patients. These results suggested that UBE2L3 might be served as a prognostic marker for NSCLC patients.

**Figure 7:**
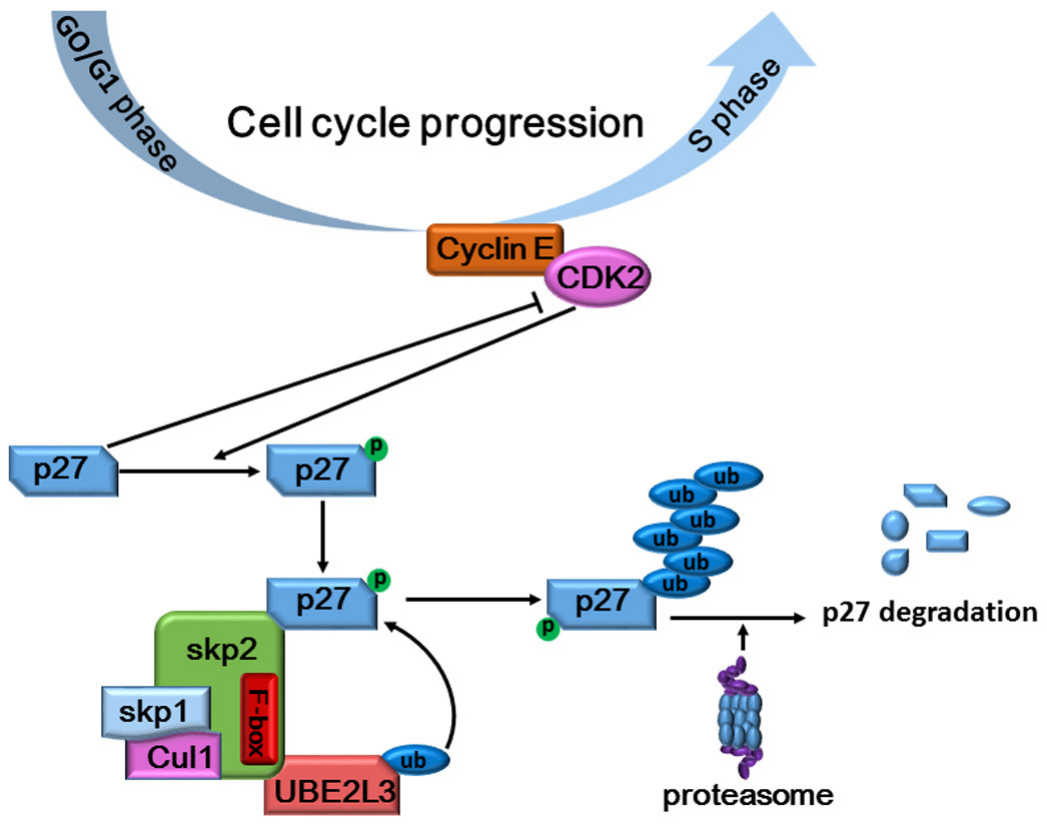
Schematic diagram summarizing how UBE2L3 regulated cell cycle through accelerating SCF (Skp2) mediated p27kip1 degradation

To investigate whether UBE2L3 was a driving factor for NSCLC carcinogenesis or not, we then evaluated the biological role of UBE2L3 in NSCLC cells by knocking down or overexpressing UBE2L3. Our results indicated that knockdown of UBE2L3 in A549 and NCI-H1299 cells significantly inhibited cell proliferation, while overexpression of UBE2L3 in NCI-H460 and HCC827 cells promoted cell growth in a cell cycle dependent manner. The cell cycle progression was mainly regulated by cyclin-dependent kinases (CDKs) and their regulatory subunits cyclins [21]. p27Kip1 is a member of CDK inhibitors (CKI), which could bind to the CDK/cyclin complex to arrest cell cycle progression [22]. Conversely, CDKs could phosphorylate CKIs for the subsequent ubiquitylation and degradation [23]. The ubiquitylation of p27Kip1 was well-reported to be executed by the SCF (Skp2), which was considered to be the largest E3 family known to date [24]. The SCF (Skp2) ubiquitin ligase complex was essential for transferring the activated E2-Ub to the lysine of target proteins. Our current study ruled out UBE2L3 as a substrate of p27kip1, a target for ubiquitylation-dependent degradation, drawing a relatively complete cascade degradation process for p27kip1 in NSCLC cells (Figure 7). Taken together, UBE2L3 controlled the ubiquitylation-dependent degradation of p27kip1 and thus affected cell cycle progression.

There still existed several limitations of the current study. Firstly, the cases enrolled in the current study were mainly from nearby areas of our hospital, and we believed that multi-center studies were still needed to confirm the clinical significance of UBE2L3. Secondly, previous literatures revealed that UBE2L3 could also act as a transcription factor and the transcriptional activity of progesterone receptor (PR), androgen and retinoic acid receptors could be affected by UBE2L3 in a hormone-dependent manner [25]. However, our current study was mainly focused on the basic roles (ubiquitin conjugating enzyme) of UBE2L3 in NSCLC cells. Despite UBE2L3 showed no transcriptional effect on p27kip1, we couldn't completely excluded the possibility that UBE2L3 could transcriptionally activate/inhibit other downstream targets to regulate cell growth or other biological behaviors including cell migration, invasion or angiogenesis. Thirdly, the latest viewpoint suggested that Yap mediated acetylation of Skp2 inhibited cell polyploidy and liver oncogenesis [26]. However, our current study didn't involve the acetylation of Skp2 in NSCLC. Whether UBE2L3 was involved in the acetylation of Skp2 still need further investigations.

In conclusion, our current work revealed that UBE2L3 was overexpressed in NSCLC tissues and cell lines. UBE2L3 regulated NSCLC cell growth in a cell-cycle dependent manner via SCF (Skp2)-mediated p27kip1 degradation. UBE2L3 and its downstream target p27kip1 might be served as novel therapeutic targets for NSCLC patients.

## MATERIALS AND METHODS

### Patients and immunohistochemical analysis

All of the human and animal experiments were approved by the Human Research Ethics Committee of The First Hospital of Jiaxing. Written consent was obtained from all participants who were fully informed about the experimental procedures during the period of research. Two independent specimen cohorts (cohort 1, n=142, from 2008-2011; cohort 2, n=28, from 2015-2016) were randomly collected from NSCLC patients underwent surgical resection. The specimens of cohort 1 were embedded with paraffin, made into tissue microarray (TMA) and stained with anti-UBE2L3 (ab108936, Abcam) or anti-p27kip1 (ab32034, Abcam) according to the manufacturer's protocol (Immunostain SP kit, DakoCytomation, USA). A positive reaction for UBE2L3/p27kip1 was scored in four grade categories according to the staining intensity (negative = 0, weak = 1, moderate = 2, strong = 3) and the percentage of UBE2L3/p27kip1-positive cells (0 = 0–1%, 1 = 1–5%, 2 = 6–29%, 3 = 30–59%, 4 = 60–100%). The final staining score were determined by the sum of the intensity and percentage scores. Therefore, each case was ultimately considered as low expression if the final score was 0 to 3 and high expression if the final score was 4 to 7. Analysis was performed by 2 independent observers who were blinded to the patient characteristics.

### Cell culture

Human NSCLC cell lines A549, NCI-H441, NCI-H460, NCI-H520, and the normal immortalized bronchial epithelial cell line HBE were purchased from American Type Culture Collection (Manassas, VA, USA). NCI-H358, NCI-H1299, NCI-H1650 and HCC827 were purchased from the Type Culture Collection of the Chinese Academy of Sciences (Shanghai, China). All cells were cultured in Dulbecco's Modified Eagle Medium (DMEM) supplemented with 10% heat-inactivated fetal bovine serum.

### Plasmid, siRNA and transient transfection

UBE2L3 expressing plasmid, p27kip1 expressing plasmid and the control plasmid were purchased from Cyagen Biosciences, USA. UBE2L3 siRNA, p27kip1 siRNA and the negative control siRNAs were purchased from GenePharma, Shanghai, China. Cells were plated at appropriate densities in a 6-well plate until reached 60 % confluence. Lipofectamine 2000 (Invitrogen, USA) was used for transient transfection according to the manufacturer's instructions. Cell viability, clonogenic and flow cytometry assays were performed 48 h after transfection.

### Quantitative real-time polymerase chain reaction

Quantitative Real-Time Polymerase Chain Reaction (qRT-PCR) was performed as previously described [27]. The primers used in the present study were UBE2L3-F, 5’-TGCCAGTCATTAGTGCTGAAAACT-3’, UBE2L3-R, 5’-GGGTCATTCACCAGTGCTATGAG-3’; p27kip1-F, 5’-TAATTGGGGCTCCGGCTAACT-3’, p27kip1-R, 5’- TGCAGGTCGCTTCCTTATTCC-3’; GAPDH-F, 5’-GC ACCGTCAAGGCTGAGAAC-3’, GAPDH-R, 5’-TGG TGAAGACGCCAGTGGA-3’.

### Cell viability and clonogenic assay

The resuspended cells were seeded in 96-well plates in triplicate at a density of 1 × 10^3^ cells per well in 200 μl of DMEM medium. Cell viability was analyzed by Cell Counting Kit-8 (CCK-8, Dojindo, Japan) assay according to the manufacturer's instructions. As to clonogenic assay, 1 × 10^3^ cells were seeded in 6-well plates and incubated until macroscopic clones formed. The clones were fixed by methanol and stained with 1 % crystal violet for counting.

### Generation of knockdown stable cells

The UBE2L3 shRNA and control shRNA were purchased from GeneChem, Shanghai, China. The shRNAs were transfected into A549/NCI-H1299 cells using lipofectamine2000 (Invitrogen, USA). Stable cell lines were obtained by continuous puromycin treatment and immunoblotting (IB) was used for identification.

### Flow cytometry assay of the cell cycle and apoptosis

Cell cycle and apoptosis were detected by flow cytometry (FACSCalibur, Becton Dickinson, MD, USA) as previously described [27].

### *In vivo* tumor growth assay

A549 and NCI-H1299 stable cells (2 × 10^6^ in 100 μl of sterilized phosphate-buffered saline) were subcutaneously injected into the dorsal flank of 4-week-old male BALB/c nude mice (five mice per group). Tumor nodules were measured every week with a caliper and the tumor volumes were calculated using the following formula: V = tumor length × tumor width^2^/2. All mice were sacrificed after 30 days and the subcutaneous tumor grafts were resected, weighed and fixed by formalin for immunohistochemical analysis.

### Immunoprecipitation and immunoblotting assay

IB assay was performed as previously described [27]. As to immunoprecipitation, cells were lysed, incubated with 2 μg corresponding antibodies overnight and incubated with protein A/G agarose for 4 h. The immunocomplexes were then resuspended with 2 × sample loading buffer and boiled for 5 min for SDS-PAGE and IB analysis. The primary antibodies used were: anti-Skp2 (ab68455, Abcam); anti-Skp1 (#12248, Cell Signaling Technology); anti-Cul1 (#4995, Cell Signaling Technology).

### Ubiquitination and protein stability assay

For ubiquitination assay, cell lysates were immunoprecipitated with anti-p27kip1, followed by IB with antibodies against ubiquitin (ab7780, Abcam). Cycloheximide (CHX, sigma) chase assay was used to determine the half-life of p27kip1. Cells were treated with CHX at the concentration of 0.1 mg/ml and harvested at the indicated time points. Then the proteins were separated by SDS-PAGE and analysed by IB.

### Statistical analysis

All data were presented as means with standard deviation. The significances of differences between two groups were assessed by two-tailed Student's t-test. The significances of differences between three groups were assessed by one-way ANOVA analysis. A *p* value less than 0.05 was considered to be significant.

## SUPPLEMENTARY MATERIALS FIGURES AND TABLES


