# Supplementary material for: Ubiquitin conjugating enzyme E2 L3 promoted tumor growth of NSCLC through accelerating p27kip1 ubiquitination and degradation
Source: Oncotarget. 2017 Aug 24;8(48):84193–203. doi: 10.18632/oncotarget.20449 (PMC5663587; doi:10.18632/oncotarget.20449)
Supplement: Supplementary file 1 [file oncotarget-08-84193-s001.pdf]

Ubiquitin conjugating enzyme E2 L3 promoted tumor growth of NSCLC through accelerating p27kip1 ubiquitination and degradation

SUPPLEMENTARY MATERIALS

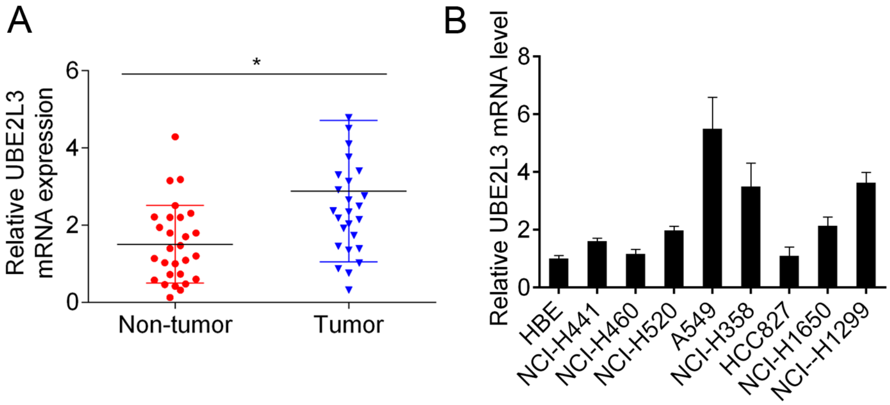

Supplementary Figure 1: (A) The mRNA levels of UBE2L3 in 28 paired NSCLC cases were evaluated by qRT-PCR assay. **(B)** The mRNA levels of UBE2L3 in eight NSCLC cell lines and the normal immortalized bronchial epithelial cell line HBE were evaluated by qRT-PCR assay.

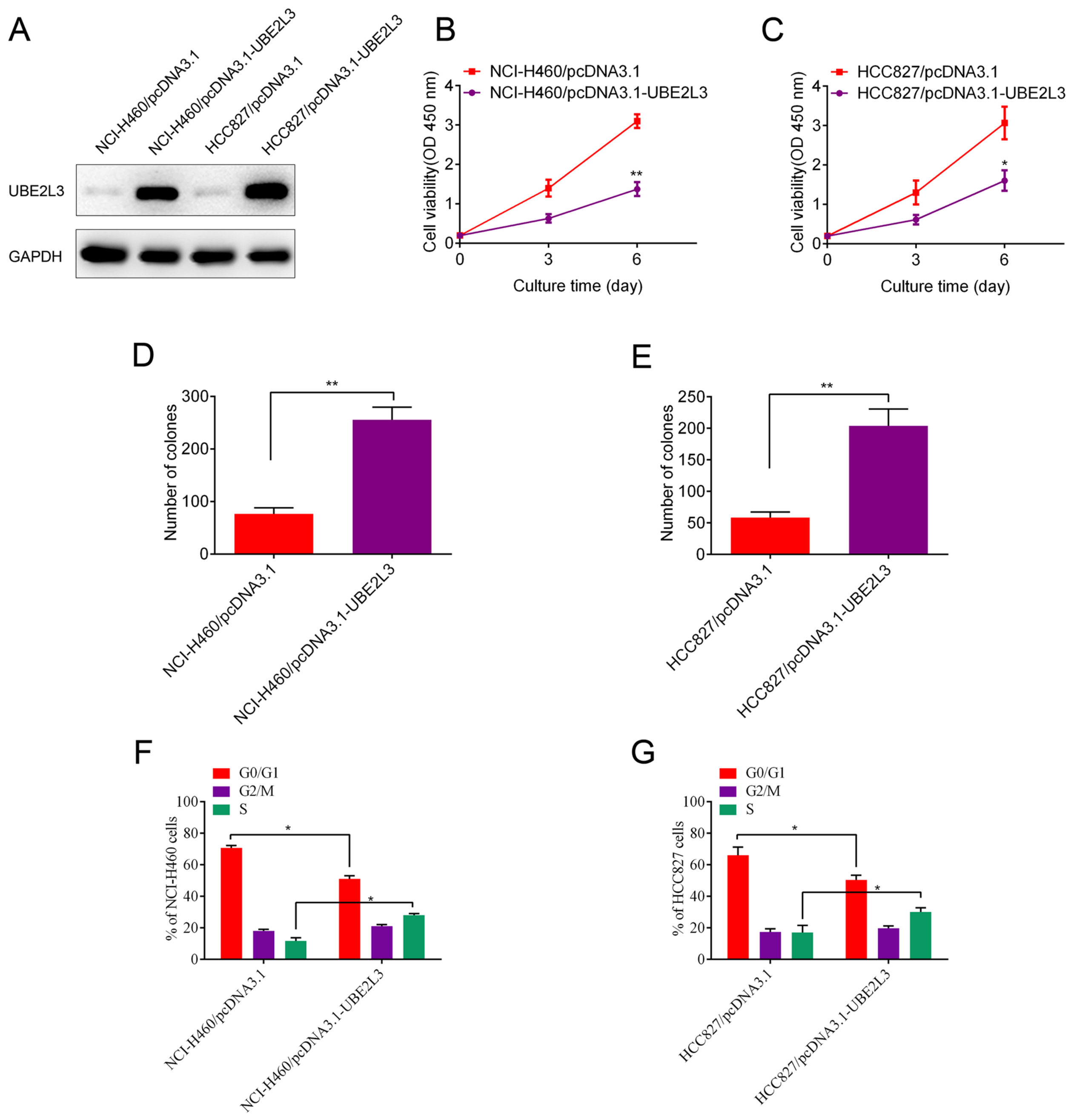

Supplementary Figure 2: (A) UBE2L3 overexpression plasmids pcDNA3. 1-UBE2L3 and the control plasmids were transfected into A549/NCI-H1299 cells and the overexpression efficiency were confirmed by IB. **(B, C)** Overexpression of UBE2L3 in NCI-H460 and HCC827 cells promoted the cell proliferation ability by CCK-8 assay. **(D, E)** Overexpression of UBE2L3 promoted the colony formation ability of NCI-H460 and HCC827 cells. **(F, G)** The results of flow cytometry showed that overexpression of UBE2L3 decreased the proportion of cells in G1 phase and increased proportion of cells in S phase.

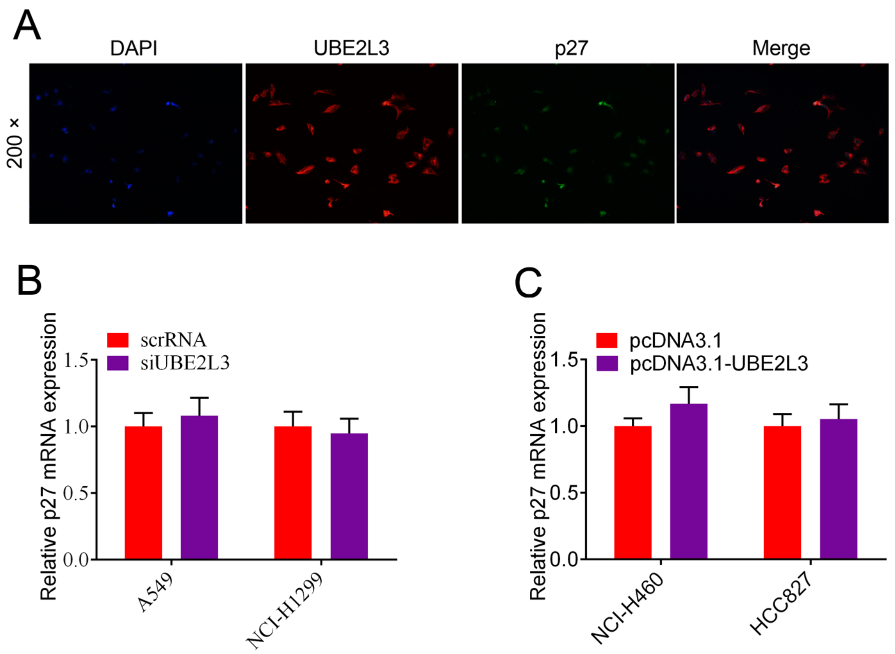

Supplementary Figure 3: (A) Co-localization of UBE2L3 and p27kip1. **(B)** The effect of UBE2L3 overexpression and down-regulation on the mRNA level p27kip1 was evaluated by qRT-PCR assay.

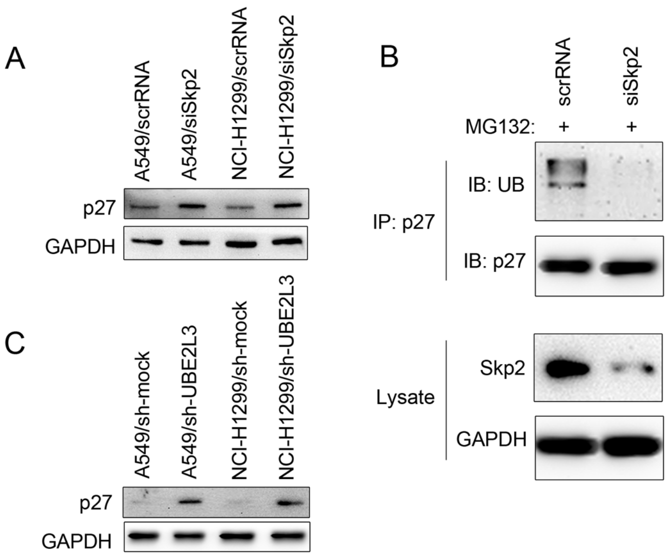

Supplementary Figure 4: (A) Knockdown of Skp2 in A549 or NCI-H1299 cells promoted the protein level of p27kip1. **(B)** Knockdown of Skp2 promoted the ubiquitination and proteasomal degradation of p27kip1. C Total proteins were extracted from subcutaneous xenografts, and the result of immunoblotting showed that knockdown of Skp2 promoted the level p27kip1 *in vivo*.
